# Comparison of Self-Report Questionnaire and Eye Tracking Method in the Visual Preference Study of a Youth–Beverage Model

**DOI:** 10.3390/foods11040505

**Published:** 2022-02-10

**Authors:** Hongbo Sun, Wanxin Wang, Xinnan Liu, Benzhong Zhu, Yue Huang, Xiaojing Leng, Lu Jia

**Affiliations:** 1 Key Laboratory of Functional Dairy, College of Food Science & Nutritional Engineering, China Agricultural University, Beijing 100083, China; b20183060491@cau.edu.cn (H.S.); xnliu0112@126.com (X.L.); zbz@cau.edu.cn (B.Z.); huangyue@cau.edu.cn (Y.H.); 2 Key Laboratory of Precision Nutrition and Food Quality, Ministry of Education, China Agricultural University, Beijing 100083, China; 3 College of Information & Electrical Engineering, China Agricultural University, Beijing 100083, China; wangwanxin97@126.com

**Keywords:** eye tracking, self-report questionnaire, food preference, visual factor

## Abstract

This study compares the characteristics of a self-report questionnaire (SRQ) and eye tracking (ET) based on a simple human–beverage visual cognition model. The young participants were mainly defined by their gender and body mass index (BMI). The beverage samples consisted of milk, coffee, cup, and coaster. SRQs allow the participants to clearly express their overall cognition of the samples in the form of vocabulary, while ET captures their hidden thinking process. The analysis, using a random forest (RF) classifier, found that participant parameters (gender and BMI) played a more important role for SRQ, while ET was related to beverage parameters (color and shape). This work reiterates that these two methods have their advantages and complement each other in food sensory analysis.

## 1. Introduction

The appearance of food can effectively affect the cognitive preferences of consumers [1,2]. The essential elements of food appearance are their color and shape, which come from their inherent nature, relative processing information, packaging materials, and environment [3,4]. Owing to the effects of physiological factors, including age, gender, and body mass index (BMI), as well as psychological, social, and cultural factors, the cognition of food appearance by consumers is generally complex [5,6,7]. The factors that can be perceived, predicted, and systematically described in conscious language are usually named as explicit types, while those that are unconscious, unpredictable, and nonverbal are implicit types [8]. Factors that aid in the comprehensive measurement of the visual cognition of consumers and understanding the impact of food appearance on their preferences are of great help in product packaging design, especially during the current epidemic period, when people travel less and often rely on the images displayed on e-commerce platforms as a shopping reference; this makes such research very valuable.

Since 1997, many methods to measure consumer cognitive features have been reported, mostly based on subjective self-reporting questionnaires (SRQs) [9]. Such operations, often based on explicit information, can obtain clear decision-making information of the consumers and also serve users conveniently and quickly. However, the gap between conscious vocabulary and unconscious thinking can easily lead to cognitive bias [10]. To overcome the limitations of language, some authors have tried to develop nonverbal food-induced emotion measurement methods to compensate for the shortcomings of the above methods [11,12,13]. Nevertheless, when the decision-making of the consumers is contextually or environmentally cue-induced, which is devoid of deliberate attitude processing and lacks rational thinking, explicit measurement is often not ideal. Therefore, implicit measurement has attracted increasing attention as an auxiliary means [8,14].

Implicit measurements do not require participants to provide their subjective reports directly. It focuses on analyzing their psychological state by measuring physiological data such as heart rate (HR), blood pressure (BP), skin temperature (ST), skin electrical activity (EDA), positron emission tomography (PET), magnetoencephalography (MEG), electroencephalography (EEG), functional magnetic resonance imaging (fMRI), and eye tracking (ET) [8,9]. A visual psychoanalysis technology, ET is based on human visual behavior research [15]. Through gaze bias theory, many authors have pointed out that gaze behavior is not only closely correlated with food choice behavior but is also actively involved in the preference formation of consumers [16,17]. ET analyzes data mainly by using the gaze of people on the visual area of interest (AOI), which is represented by the visual hotspots distributed on the object image projected on a computer screen [15]. In most software, gaze intensity, from high to low, is color-coded [18]. Understanding the difference between ET and SRQ is important for improving visual sensory analysis technology.

In this study, the human–food visual cognitive model for comparing the explicit and implicit measures is based on a traditional stimulus–organism–response (S-O-R) paradigm. This paradigm was first proposed by Mehrabian and Russell [19] and later improved by Lin [20], Bitner [21], and Schreuder et al. [22]. According to their interpretation, stimulus mainly refers to the physical stimulation of the human body by food, organism mainly refers to the early cognitive stage of humans, and response mainly refers to the conscious expression of language when people have enough cognition of the stimulus. This paradigm can concisely associate human parameters with food parameters. Accordingly, the participants’ parameters were mainly classified as gender and BMI in this study. The visual parameters of food samples, that is, color and shape, were mainly provided by specific paper cups and coasters presented during the beverage model (milk and coffee) measurements. The SRQ and ET were used to measure the visual preferences of participants, and they represented the explicit and implicit methods, respectively. Correspondence analysis (CA) and random forest (RF) classification analysis were used to analyze the parameter correlations between the participants and the food samples. The main purpose of this manuscript is to compare the differences between SRQ and ET methods through the study of a specific human–food model so as to further aid in the comprehensive measurement of the visual cognition of consumers and understand the impact of food appearance on their preferences, which will further innovate product packaging design.

## 2. Materials and Methods

### 2.1. Participants

In total, 78 people participated in this study (38 men and 40 women). Among them, there were 28 normal-weight (NW, BMI = 18.5–24.9), 26 underweight (UW, BMI < 18.5), and 24 overweight (OW, BMI > 25) participants. The participants were undergraduates majoring in food science, and their mean age was 21 ± 0.7 years. Their sensory abilities, including taste, smell, and vision, were normal, and none were allergic to milk or coffee. This study was approved by the Medical Ethical Committee of China Agricultural University (Project identification code: CAUHR-2019011).

### 2.2. Visual Samples

**Figure 1:**
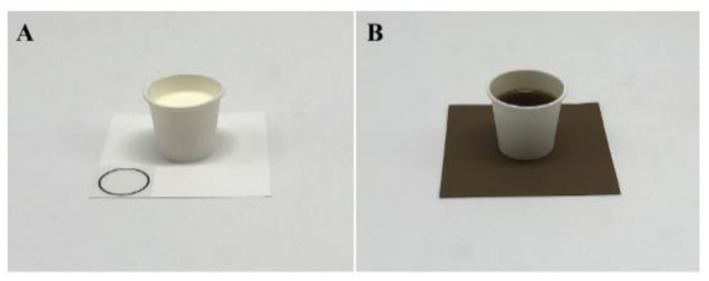
Combination of the representative visual samples presented to participants. (**A**) a combination of milk with a circle; (**B**) a combination of coffee with a brown coaster.

Milk, coffee, paper cups (white, 25 mL), and square coasters (5 colors, 9 × 9 cm) were purchased from the supermarket. The five coaster colors included *white* (H (hue): 0°, S (saturation): 0%, L (lightness): 100%), *blue* (H: 210°, S: 100%, L: 60%), *green* (H: 120°, S: 50%, L: 60%), *brown* (H: 40°, S: 100%, L: 30%), and *red* (H: 0°, S: 60%, L: 50%). The five geometric shapes used in this work were: *circle* (diameter 3 cm), *hexagon* (side length 1.5 cm), *pentagon* (side length 1.8 cm), *square* (side length 3 cm), and *triangle* (side length 3 cm). These geometric shapes were pasted on the lower-left corner of the white coaster with transparent self-adhesives. The combination of beverages (milk and coffee) with these coasters formed 20 visual samples (two of the samples are illustrated in Figure 1). The combined samples were randomly presented to the participants one at a time.

### 2.3. Description Evaluation

The participants were asked to observe different colors (i.e., *white*, *blue*, *green*, *brown*, and *red*) and shapes (i.e., *circle*, *hexagon*, *pentagon*, *square*, and *triangle*) that were used in each of the two beverages scenes, respectively. Then, they were requested to describe each stimuli using simple words (descriptors). CA is a technique for sensory evaluation; its data are widely used to visualize a contingency table to obtain sample and descriptors configurations in the form of points in orthogonal space [23]. The frequency of descriptors used was counted for further CA.

### 2.4. SRQ Operation

The participants used the 3-point liking scoring method (1 = low, 2 = medium, 3 = high) to express their preferences for the above samples [24]. The reason for using the 3-point liking scoring method in this study was that it was easy to obtain obvious decisions from participants.

### 2.5. ET Operation

**Figure 2:**
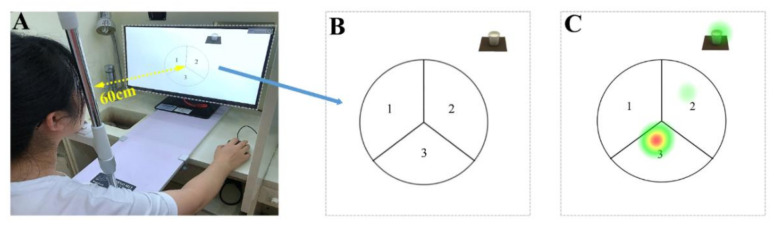
Eye-tracking measurement. (**A**) Participant-device position. (**B**) Slide displayed on the screen. (**C**) Visual hotspot map on the screen.

The ET measurement, using an EyeSo Ec-80 eye tracker (60 Hz) (Brain craft Technology Co., Ltd., Beijing, China), was performed after SRQ measurement. Calibration was performed using a 9-point calibration procedure provided with EyeSo Studio software version 3.3 (Brain craft Technology Co., Ltd., Beijing, China), and the participants were asked to keep their heads still during the experiment [24]. The distance between the eyes of the participants and the screen (21″ full HD, resolution: 1920 × 1080 pixels) was fixed at 60 cm with a head bracket (Figure 2A). As a scoring reference, the sample image was displayed on the same slide on the screen along with the scoring table (Figure 2B). The participants rated the samples by observing the scoring table to express their preferences for the above samples without subjective reports. Before the ET measurement, the participants underwent operation training. During the formal measurement, the participants were required to continuously observe 20 scoring reference slides in random order. Between two consecutive slides, a cross cursor at the screen center for 2 s was used to maintain the calibration by visual inspection [25]. The observation time of every scoring reference slide for each slide was 7 s [26], and this time control was designed to avoid visual fatigue caused by watching the screen for a longer time.

### 2.6. Data Processing

#### 2.6.1. Correspondence Analysis

Based on the chi-square distance, CA on word frequency was carried out using the SPSS statistical package [24] (SPSS 22.0, IBM Corporation, Armonk, NY, USA).

#### 2.6.2. ET Gaze Processing

The scoring table was designed as trisection circular visual AOI, as shown in Figure 2B, where each sector corresponded to a certain score (AOI1 = 1 = low, AOI2 = 2 = medium, and AOI3 = 3 = high). The gaze intensity, recorded as the dwell time by ET, indicated the participants’ attention to the relative score. The gaze intensity< from high to low, was represented by red through yellow to green colors [18]. With a longer dwell time, the gaze intensity increases, and the score becomes more definite. Figure 2C shows the ET hotspot map of the sample, with a score of 3.

#### 2.6.3. Random Forest Classifier

The RF classifier is an integrated machine learning algorithm extended from a decision tree (DT) with superior predictive performance, and it further analyzes the weights of the prediction factors in the model [27]. According to the method proposed by Deng et al. [27], the RF classifier code was written using Python v.3.7 (Anaconda, Inc., Austin, TX, USA), with slight modifications. This code embeds the datasets in a Euclidean space through one-hot coding and normalization preprocessing. The participant parameters, that is, gender (male or female) and BMI (NW, UW, or OW), were digitized with 1 or 0 (yes or no) for each person, respectively. This code constructed 100 classification trees using a bootstrap strategy, using randomly selected training sample subsets and predictors for ternary splits. A default impurity, Gini, was used to determine the splitting quality. The code performance was empirically evaluated using 10-fold cross-validation. The hyperparameters in the code were automatically adjusted by grid-searching cross-validation. The code prediction accuracy was estimated by averaging the metrics of the 10 groups.

### 2.7. Statistical Analysis

Statistical data were analyzed using paired *t*-tests to understand the differences between SRQ and ET measures. Analysis of variance (ANOVA) was used to obtain consumer preferences for visual samples, and Bonferroni’s multiple comparisons test (*p* < 0.05) was conducted using GraphPad Prism 7.04 (GraphPad, Inc., San Diego, CA, USA).

## 3. Results

### 3.1. Correspondence analysis

**Figure 3:**
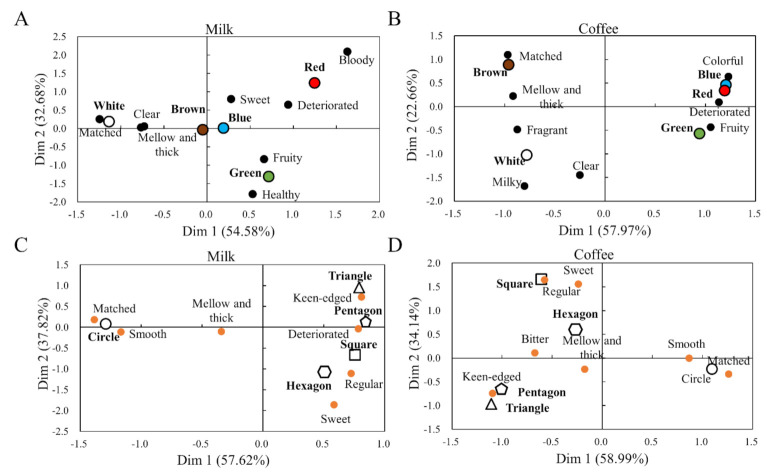
Correspondence analysis between color or shape stimuli and descriptors in the different beverage systems. The *x*−axis represents the first dimension (Dim 1), while the *y*−axis represents the second dimension (Dim 2). (**A**) Color preferences of milk; (**B**) color preferences of coffee; (**C**) shape preferences of milk; (**D**) shape preferences of coffee.

Figure 3 compares the correspondence between the color (Figure 3A,B) or shape stimuli (Figure 3C,D) and descriptors in the different beverage systems (Figure 3A,C for milk, Figure 3B,D for coffee). The distance between the word points in the figure can be used as a reference for comparing their correspondence level; the shorter the distance, the stronger the correspondence [28].

In the color part, CA denoted 87.26% of the milk variance (Dim 1 = 54.58%, Dim 2 = 32.68%) (Figure 3A) and 80.63% of the coffee variance (Dim 1 = 57.97%, Dim 2 = 22.66%) (Figure 3B). The closest distance between the color stimuli and descriptors was *white*-*matched* in milk and *brown*-*matched* in coffee. *White* in milk and *brown* in coffee were also close to *mellow and thick*, respectively. The spatial proximities of *white*-*clear*, *green*-*fruity*, and *red*-*deteriorated* in both beverages were similar. The correspondence of the other words is less obvious.

In the shape part, CA denoted 95.44% of the milk variance (Dim 1 = 57.62%, Dim 2 = 37.82%) (Figure 3C) and 93.13% of the coffee variance (Dim 1 = 58.99%, Dim 2 = 34.14%) (Figure 3D). The closest distance between the shape stimuli and descriptors was *circle-matched* and *circle*-*smooth* in milk and *circle*-*matched* and *circle*-*smooth* in coffee. The spatial proximities of *triangle*-*keen*-*edged* and *pentagon*-*keen*-*edged* were similar in both beverages. The correspondence of other words was not easy to distinguish.

### 3.2. Preferences of the Whole Group

**Figure 4:**
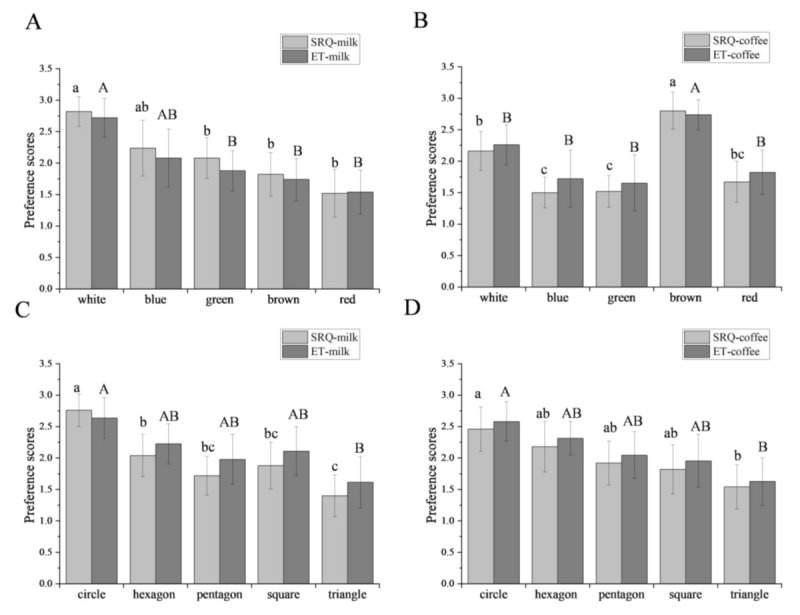
Comparison of the visual preferences of milk and coffee by self-report questionnaire (SRQ) and eye-tracking (ET) measurements. Different lowercase letters mark significant differences in the SRQ-beverage model; Different uppercase letters mark significant differences in the ET-beverage model. (**A**) Color preferences of milk; (**B**) color preferences of coffee; (**C**) shape preferences of milk; (**D**) shape preferences of coffee.

Figure 4 compares the preference scores of the whole group obtained using the SRQ and ET measurements. Figure 4A and 4B show the color effects of the coasters on milk and coffee, and Figure 4C and 4D are related to the shape effects of the coasters on milk and coffee, respectively. In the color part for both SRQ and ET, *white* in milk, and *brown* in coffee exhibited the highest preference, while *red* in milk, as well as *green* and *blue* in coffee, exhibited lower preference. In the shape part, only *circles* caused a higher preference for both beverages. The results of the two methods are consistent in all cases.

### 3.3. Preferences of the Gender Groups

**Figure 5:**
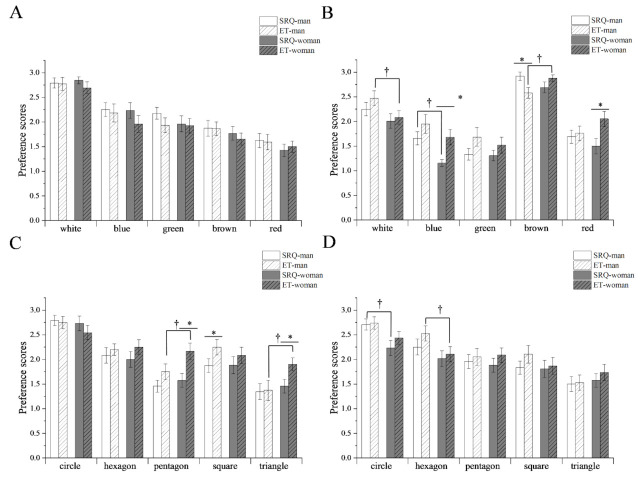
Comparison of the preferences scores of the self-report questionnaire (SRQ) and eye-tracking (ET) measure of the gender groups. † Indicates statistical significance at *p* < 0.05 (†) between males and females. * Indicates statistical significance at *p* < 0.05 (*) between SRQ and ET. (**A**) Color preferences of milk; (**B**) color preferences of coffee; (**C**) shape preferences of milk; (**D**) shape preferences of coffee.

Figure 5 compares the preference scores of the gender groups obtained using the SRQ and ET measurements. Figure 5A,B show the color effects of the coasters on milk and coffee, and Figure 5C,D show the shape effects of the coasters on milk and coffee, respectively. In terms of color, there were significant differences in gender preferences for *white*, *blue*, and *brown* coffee. The results of SRQ and ET for *blue*, *brown*, and *red* coffee were inconsistent. These significant differences were not observed in milk. In terms of shape, there were significant differences in gender preferences for *pentagon* and *triangle* in milk and *circle* and *hexagon* in coffee. The results of SRQ and ET were consistent in coffee but inconsistent for *pentagon*, *square*, and *triangle* in milk. Although gender had a certain impact on the preferred choice, the combinations of milk-*white*, milk-*circle*, coffee-*brown*, and coffee-*circle* had the highest preference (Supplementary Materials, Tables S1–S4).

### 3.4. Preferences of the BMI Groups

**Figure 6:**
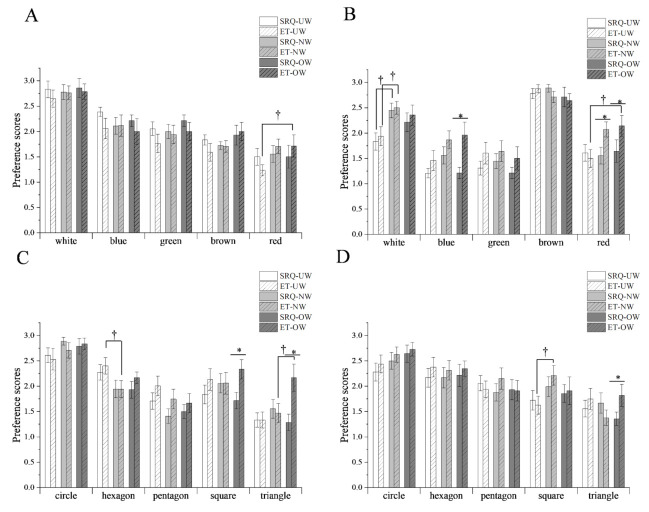
Comparison of the preferences scores of the self-report questionnaire (SRQ) and eye-tracking (ET) measure of the BMI groups. UW: underweight; NW: normal weight; OW: overweight. † Indicates statistical significance at *p* < 0.05 (†) between body mass index (BMI) groups. * Indicates statistical significance at *p* < 0.05 (*) between SRQ and ET. (**A**) Color preferences of milk; (**B**) color preferences of coffee; (**C**) shape preferences of milk; (**D**) shape preferences of coffee.

Figure 6 compares the preference scores of the BMI groups obtained from the SRQ and ET measurements. Figure 6A,B show the color effects of the coasters on milk and coffee, and Figure 6C,D are related to the shape effects of the coasters on milk and coffee, respectively.

In terms of color, there were significant differences in BMI preferences for *red* in milk and *white* and *red* in coffee. The results of SRQ and ET were consistent in milk but inconsistent for *blue* and *red* in coffee. In terms of shape, there were significant differences in BMI preferences for *hexagon* and *triangle* in milk and *square* in coffee. The results of SRQ and ET for *square* and *triangle* in milk and *triangle* in coffee were inconsistent. Similarly, BMI analysis also showed that the combinations of the milk-*white*, milk-*circle*, coffee-*brown*, and coffee-*circle* had the highest preference (Supplementary Materials, Tables S5–S8).

### 3.5. Random Forest Classifier Analysis

Table 1 compares the SRQ and ET analysis of the combined beverage samples with the highest preference using the RF classifier. The datasets of these samples were named WCM-ET (*white*-*circle*-milk-ET), WCM-SRQ (*white*-*circle*-milk-SRQ), BCC-ET (*brown*-*circle*-coffee-ET), and BCC-SRQ (*brown*-*circle*-coffee-SRQ), respectively. These datasets contained the information of the participants (gender and BMI) and beverage samples (color, i.e., *white* or *brown*, and shape, i.e., *circle*). The prediction accuracies varied between 67.2–76.4% in all cases. In SRQ, the total contributions of participants (0.72 in milk and 0.59 in coffee) were higher than those of beverage samples (0.28 in milk and 0.41 in coffee), while in ET, the total contributions of participants (0.25 in milk and 0.39 in coffee) were lower than those of beverage samples (0.75 in milk and 0.61 in coffee).

## 4. Discussion

The CA was used to preliminarily investigate the cognitive status of the participants in the beverage samples (various combinations of beverages, paper cups, and coasters). According to the experimental design, the visual characteristics of these samples could be mainly covered by two groups of stimuli words, colors (*red, green, blue, brown, black,* and *white*) and shapes (*circle, hexagon, pentagon, square,* and *triangle*). It was expected that the correspondence between these stimuli samples and the descriptors freely provided by the participants (such as *matched, mellow and thick, milky, fruity, smooth, keen-edged*) could reflect their cognitive state.

In Figure 3, the descriptor *matched* was very close to *white* or *circle* of milk as well as *brown* or *circle* of coffee, indicating that the participants had a consistent recognition of the collocation of the *white*-*circle*-milk and *brown*-*circle*-coffee. Such thinking usually evolves from the common sense of participants and several experiences of their explicit or implicit life. In contrast, other descriptors lacked this matching cognition; for example, *red* was closer to *bloody*, *green* was closer to *fruity*, *triangle* was closer to *keen-edge*, and so on, but “*matched*” was far from them in the figures.

SRQ and ET methods were used to further analyze the preferences of the above samples, respectively. Both the methods found that the participants in the whole group (Figure 4), gender groups (Figure 5), or BMI groups (Figure 6) had the highest preference for the combinations of *white*-milk, *circle*-milk, *brown*-coffee, and *circle*-coffee. This finding indicates that the word *matched* in Figure 3 has high preference characteristics.

In contrast, for other samples, it was difficult to make the conclusions of these two methods consistently under the above grouping conditions. Furthermore, many studies have inferred that the appearance of products can interfere with the preference behavior of the consumer, and the gender, weight, and health status of the consumer can also affect their living habits and food preferences, which deviate them from real ideas and aid in making choices conducive to themselves [29,30,31]. In this context, consumer preferences are often complex, ambiguous, and difficult to capture correctly.

Considering that the focus of this work was to investigate the differences between SRQ and ET, the samples that led to the inconsistency between these two methods were temporarily avoided. *White*-milk, *circle*-milk, *brown*-coffee, and *circle*-coffee, as the combination research objects (WCM and BCC), were used with the RF classifier to process the relevant datasets.

The RF classifier is a common machine-learning classifier. When dealing with nonlinear multi-parameter relationships, it can exhibit the contribution of each parameter through weight analysis [27,32]. Table 1 compares the weights of the participant parameters (BMI and gender) and the sample visual parameters (color and shape) in the SRQ and ET measures, respectively. In both milk and coffee, the weight of the participant parameters was higher than that of the sample visual parameters in the SRQ, but the opposite was true for ET. In the SRQ operation, the participants could immediately express their decisions by selecting the scores on the questionnaire. As mentioned earlier, under conscious control, their physical state may participate in their cognitive processes and eventually produce deliberate operations [29,30,33]. Conversely, there was no need to immediately express the decision in ET measurement, so the visual traces left by the participants on the computer screen included the conscious decision information and the unconscious thinking information stimulated by images [34,35]. Moreover, their final decisions were determined by the experimenters and not by themselves, which might be the main reason underlying the differences between the SRQ and ET measurements.

Briefly, the participants’ explicit and implicit thinking would be intertwined, which would be reflected in the data of SRQ and ET. However, the difference in measurement principles led to the respective advantages of the two methods. The former allowed the participants to clearly express their overall cognition of the samples in the form of vocabulary, represented by the CA diagram, while the latter could capture their hidden thinking process. Finally, by comparing SRQ and ET data, RF showed the implicit thinking characteristics of the participants through the influence weight of the various factors. It should also be noted that the accuracy of the RF classifier was only between 67.2–76.4%, which indicated that the present human–food model and the two methods may have systematic errors in the selection of the color/shape stimuli and descriptors (degree of vocabulary optimization), control over the ability of the participants (their level of free expression and observation), and even environmental impact (e.g., interference from color and shape visual factors of the laboratory furniture and equipment). In addition, the selection of specific participants and samples inevitably limits the universality of the conclusions. These details warrant further study.

## 5. Conclusions

In this study, the effects of participants and beverages on SRQ and ET were compared by analyzing a simple human–beverage visual model. The results show that the beverage appearance factors (color and shape) affected the participants’ preferences, while the physiological factors of participants (gender and BMI) also affected their preferences. Through the analysis of RF classifiers, it was noted that the influence of beverage appearance played a greater role in ET measurement. In comparison, the influence of the physiological factors of participants played a greater role in SRQ measurement. The different characteristics of the two methods indicate that they can complement each other in sensory research.

## Figures and Tables

**Table 1:** Comparison of the relative contributions of the participants’ parameters and visual parameters using the RF classifier, as well as the accuracy of prediction datasets of WCM-ET, WCM-SRQ, BCC-ET, and BCC-SRQ.

Datasets		Prediction Datasets Accuracy (%)	Participant Parameter Contribution			Visual Parameter Contribution		
			BMI	Gender	Sum	Color	Shape	Sum
Milk	WCM-ET	74.3	0.16	0.09	0.25	0.29	0.46	0.75
	WCM-SRQ	76.4	0.50	0.22	0.72	0.19	0.09	0.28
Coffee	BCC-ET	67.3	0.33	0.06	0.39	0.19	0.42	0.61
	BCC-SRQ	67.2	0.47	0.12	0.59	0.17	0.24	0.41

WCM-ET: *white*-*circle*-milk-ET; WCM-SRQ: *white*-*circle*-milk-SRQ; BCC-ET: *brown*-*circle*-coffee-ET; BCC-SRQ: *brown*-*circle*-coffee-SRQ.

## Data Availability

Not applicable.

## Supplementary Materials

The following supporting information can be downloaded at: https://www.mdpi.com/article/10.3390/foods11040505/s1, Table S1: The color preferences scores of SRQ or ET measurements of different gender groups in milk samples. Table S2: The color preferences scores of SRQ or ET measurements of different gender groups in coffee samples. Table S3: The shape preferences scores of SRQ or ET measurements of different gender groups in milk samples. Table S4: The shape preferences scores of SRQ or ET measurements of different gender groups in coffee samples. Table S5: The color preferences scores of SRQ or ET measurements of different BMI groups in milk samples. Table S6: The color preferences scores of SRQ or ET measurements of different BMI groups in coffee samples. Table S7: The shape preferences scores of SRQ or ET measurements of different BMI groups in milk samples. Table S8: The shape preferences scores of SRQ or ET measurements of different BMI groups in coffee samples.

## References

[1] Jaeger S.R., Xia Y., Le Blond M., Beresford M.K., Hedderley D.I., Cardello A.V. (2019). Supplementing hedonic and sensory consumer research on beer with cognitive and emotional measures, and additional insights via consumer segmentation. Food Qual. Prefer..

[2] Laureati M., Sandvik P., Almli V.L., Sandell M., Zeinstra G.G., Methven L., Wallner M., Jilani H., Alfaro B., Proserpio C. (2020). Individual differences in texture preferences among European children: Development and validation of the Child Food Texture Preference Questionnaire (CFTPQ). Food Qual. Prefer..

[3] Spence C., Velasco C. (2018). On the multiple effects of packaging colour on consumer behaviour and product experience in the ‘food and beverage’ and ‘home and personal care’ categories. Food Qual. Prefer..

[4] Nyitrai Á., Urbin Á., Nagy B.V., Sipos L. (2022). Novel approach in sensory color masking: Effects of colored environments on chocolates with different cocoa content. Food Qual. Prefer..

[5] Moller A., Elliot A., Maier M. (2009). Basic Hue-Meaning Associations. Emotion.

[6] Spence C. (2015). On the psychological impact of food colour. Flavour.

[7] Werthmann J., Jansen A., Roefs A. (2014). Worry or craving? A selective review of evidence for food-related attention biases in obese individuals, eating-disorder patients, restrained eaters and healthy samples. Proc. Nutr. Soc..

[8] Lagast S., Gellynck X., Schouteten J.J., De Herdt V., De Steur H. (2017). Consumers’ emotions elicited by food: A systematic review of explicit and implicit methods. Trends Food Sci. Technol..

[9] Kaneko D., Toet A., Brouwer A.-M., Kallen V., Erp J. (2018). Methods for Evaluating Emotions Evoked by Food Experiences: A Literature Review. Front. Psychol..

[10] Morin C. (2011). Neuromarketing: The New Science of Consumer Behavior. Society.

[11] Dalenberg J.R., Gutjar S., ter Horst G.J., de Graaf K., Renken R.J., Jager G. (2014). Evoked Emotions Predict Food Choice. PLoS ONE.

[12] de Wijk R.A., Noldus L.P.J.J. (2021). Using implicit rather than explicit measures of emotions. Food Qual. Prefer..

[13] Ismael D., Ploeger A. (2019). Development of a Sensory Method to Detect Food-Elicited Emotions Using Emotion-Color Association and Eye-Tracking. Foods.

[14] Dijksterhuis A., Bos M.W., Nordgren L.F., van Baaren R.B. (2006). On making the right choice: The deliberation-without-attention effect. Science.

[15] Motoki K., Saito T., Onuma T. (2021). Eye-tracking research on sensory and consumer science: A review, pitfalls and future directions. Food Res. Int..

[16] Orquin J.L., Mueller Loose S. (2013). Attention and choice: A review on eye movements in decision making. Acta Psychol..

[17] Shimojo S., Simion C., Shimojo E., Scheier C. (2003). Gaze bias both reflects and influences preference. Nat. Neurosci..

[18] Piqueras-Fiszman B., Velasco C., Salgado-Montejo A., Spence C. (2013). Using combined eye tracking and word association in order to assess novel packaging solutions: A case study involving jam jars. Food Qual. Prefer..

[19] Mehrabian A., Russell J.A. (1974). An Approach to Environmental Psychology.

[20] Lin I.Y. (2004). Evaluating a servicescape: The effect of cognition and emotion. Int. J. Hosp. Manag..

[21] Bitner M.J. (1992). Servicescapes: The Impact of Physical Surroundings on Customers and Employees. J. Mark..

[22] Schreuder E., van Erp J., Toet A., Kallen V.L. (2016). Emotional Responses to Multisensory Environmental Stimuli: A Conceptual Framework and Literature Review. SAGE Open.

[23] Vidal L., Tárrega A., Antúnez L., Ares G., Jaeger S.R. (2015). Comparison of Correspondence Analysis based on Hellinger and chi-square distances to obtain sensory spaces from check-all-that-apply (CATA) questions. Food Qual. Prefer..

[24] Antúnez L., Machín L., Ares G., Jaeger S.R. (2019). Visual attention to rate-all-that-apply (RATA) questions: A case study with apple images as food stimuli. Food Qual. Prefer..

[25] Manippa V., van der Laan L.N., Brancucci A., Smeets P.A.M. (2019). Health body priming and food choice: An eye tracking study. Food Qual. Prefer..

[26] Goyal S., Miyapuram K.P., Lahiri U. Predicting Consumer’s Behavior Using Eye Tracking Data. Proceedings of the 2015 Second International Conference on Soft Computing and Machine Intelligence (ISCMI).

[27] Deng X., Liu Z., Zhan Y., Ni K., Zhang Y., Ma W., Shao S., Lv X., Yuan Y., Rogers K.M. (2020). Predictive geographical authentication of green tea with protected designation of origin using a random forest model. Food Control.

[28] Jaeger S.R., Beresford M.K., Paisley A.G., Antúnez L., Vidal L., Cadena R.S., Giménez A., Ares G. (2015). Check-all-that-apply (CATA) questions for sensory product characterization by consumers: Investigations into the number of terms used in CATA questions. Food Qual. Prefer..

[29] Tepper B.J., Banni S., Melis M., Crnjar R., Tomassini Barbarossa I. (2014). Genetic sensitivity to the bitter taste of 6-n-propylthiouracil (PROP) and its association with physiological mechanisms controlling body mass index (BMI). Nutrients.

[30] Mora M., Urdaneta E., Chaya C. (2018). Emotional response to wine: Sensory properties, age and gender as drivers of consumers’ preferences. Food Qual. Prefer..

[31] Yarar N., Machiels C.J.A., Orth U.R. (2019). Shaping up: How package shape and consumer body conspire to affect food healthiness evaluation. Food Qual. Prefer..

[32] Gromski P.S., Correa E., Vaughan A.A., Wedge D.C., Turner M.L., Goodacre R. (2014). A comparison of different chemometrics approaches for the robust classification of electronic nose data. Anal. Bioanal. Chem..

[33] Vadiveloo M., Principato L., Morwitz V., Mattei J. (2019). Sensory variety in shape and color influences fruit and vegetable intake, liking, and purchase intentions in some subsets of adults: A randomized pilot experiment. Food Qual. Prefer..

[34] Yasui Y., Tanaka J., Kakudo M., Tanaka M. (2019). Relationship between preference and gaze in modified food using eye tracker. J. Prosthodont. Res..

[35] Sung Y.-C., Tang D.-L. (2007). Unconscious processing embedded in conscious processing: Evidence from gaze time on Chinese sentence reading. Conscious. Cogn..

