# Supplementary material for: Comparison of Self-Report Questionnaire and Eye Tracking Method in the Visual Preference Study of a Youth–Beverage Model
Source: Foods. 2022 Feb 10;11(4):505. doi: 10.3390/foods11040505 (PMC8870819; doi:10.3390/foods11040505)
Supplement: Supplementary file 1 [file foods-11-00505-s001.zip › foods-1567384-supplementary.pdf]

Table S1. The color preferences scores of SRQ or ET measurements of different gender groups in milk samples.

Stimulus	SRQ-man	ET-man	SRQ-woman	ET-woman
white	2.79±0.1 ^{a}	2.77±0.13^{a}	2.85±0.07^{a}	2.69±0.12^{a}
blue	2.25±0.14^{b}	2.18±0.18^{b}	2.23±0.16^{b}	1.96±0.17^{b}
green	2.18±0.12^{b}	1.93±0.15^{bc}	1.96±0.16^{bc}	1.92±0.15^{b}
brown	1.88±0.16^{c}	1.86±0.14^{c}	1.77±0.14^{c}	1.65±0.12^{c}
red	1.63±0.15^{d}	1.59±0.16^{d}	1.42±0.13^{d}	1.5±0.11^{c}

Different letters within the same column indicate significant difference ($P < 0.05$). SRQ: self-report questionnaire; ET: eye tracking.

Table S2. The color preferences scores of SRQ or ET measurements of different gender groups in coffee samples.

Stimulus	SRQ-man	ET-man	SRQ-woman	ET-woman
white	2.25±0.14^{b}	2.47±0.15^{ab}	2.01±0.15^{b}	2.08±0.14^{b}
blue	1.66±0.13^{c}	1.95±0.19^{cd}	1.15±0.07^{d}	1.68±0.16^{c}
green	1.33±0.12^{d}	1.68±0.19^{d}	1.31±0.11^{cd}	1.52±0.16^{c}
brown	2.92±0.08^{a}	2.58±0.11^{a}	2.69±0.11^{a}	2.88±0.07^{a}
red	1.69±0.13^{c}	1.76±0.15^{d}	1.5±0.15^{c}	2.05±0.15^{b}

Different letters within the same column indicate significant difference ($P < 0.05$). SRQ: self-report questionnaire; ET: eye tracking.

Table S3. The shape preferences scores of SRQ or ET measurements of different gender groups in milk samples.

Stimulus	SRQ-man	ET-man	SRQ-woman	ET-woman
circle	2.79±0.1^{a}	2.75±0.12^{a}	2.73±0.15^{a}	2.54±0.14^{a}
hexagon	2.08±0.16^{b}	2.2±0.12^{b}	2±0.16^{b}	2.25±0.15^{b}
pentagon	1.46±0.12^{d}	1.75±0.16^{c}	1.58±0.14^{c}	2.17±0.16^{bc}
square	1.88±0.14^{c}	2.25±0.16^{b}	1.88±0.18^{b}	2.08±0.17^{bc}
triangle	1.35±0.16^{d}	1.38±0.2^{d}	1.46±0.14^{c}	1.9±0.13^{c}

Different letters within the same column indicate significant difference ($P < 0.05$). SRQ: self-report questionnaire; ET: eye tracking.

Table S4. The shape preferences scores of SRQ or ET measurements of different gender groups in coffee samples.

Stimulus	SRQ-man	ET-man	SRQ-woman	ET-woman
circle	2.71±0.11^{a}	2.74±0.13^{a}	2.23±0.15^{a}	2.43±0.13^{a}
hexagon	2.25±0.16^{b}	2.53±0.16^{ab}	2.02±0.16^{ab}	2.1±0.15^{b}
pentagon	1.96±0.14^{c}	2.05±0.17^{c}	1.88±0.14^{b}	2.09±0.15^{b}
square	1.83±0.13^{c}	2.11±0.18^{c}	1.81±0.18^{b}	1.87±0.17^{bc}
triangle	1.5±0.15^{d}	1.53±0.16^{d}	1.58±0.14^{c}	1.74±0.16^{c}

Different letters within the same column indicate significant difference ($P < 0.05$). SRQ: self-report questionnaire; ET: eye tracking.

Table S5. The color preferences scores of SRQ or ET measurements of different BMI groups in milk samples.

Stimulus	SRQ-UW	SRQ-NW	SRQ-OW	ET-UW	ET-NW	ET-OW
white	$2.83±0.17^{a}$	$2.78±0.15^{a}$	$2.86±0.19^{a}$	$2.65±0.17^{a}$	$2.76±0.14^{a}$	$2.79±0.15^{a}$
blue	$2.39±0.09^{b}$	$2.11±0.17^{b}$	$2.21±0.11^{b}$	$2.06±0.2^{b}$	$2.12±0.21^{b}$	$2±0.26^{b}$
green	$2.06±0.14^{c}$	$2±0.15^{b}$	$2.21±0.11^{b}$	$1.76±0.18^{c}$	$1.94±0.18^{b}$	$2±0.18^{b}$
brown	$1.83±0.1^{d}$	$1.72±0.08^{c}$	$1.93±0.19^{c}$	$1.59±0.17^{c}$	$1.71±0.11^{c}$	$2±0.18^{b}$
red	$1.5±0.16^{e}$	$1.56±0.17^{d}$	$1.5±0.23^{d}$	$1.24±0.11^{d}$	$1.71±0.14^{c}$	$1.71±0.22^{c}$

Different letters within the same column indicate significant difference ($P < 0.05$). SRQ: self-report questionnaire; ET: eye tracking; BMI: body mass index.

Table S6. The color preferences scores of SRQ or ET measurements of different BMI groups in coffee samples.

Stimulus	SRQ-UW	SRQ-NW	SRQ-OW	ET-UW	ET-NW	ET-OW
white	$1.83±0.17^{b}$	$2.44±0.15^{b}$	$2.21±0.19^{b}$	$1.94±0.19^{b}$	$2.5±0.13^{b}$	$2.36±0.2^{b}$
blue	$1.21±0.09^{c}$	$1.56±0.17^{c}$	$1.21±0.11^{d}$	$1.46±0.2^{c}$	$1.87±0.18^{d}$	$1.96±0.26^{c}$
green	$1.31±0.14^{c}$	$1.44±0.15^{c}$	$1.21±0.11^{d}$	$1.61±0.21^{c}$	$1.64±0.2^{de}$	$1.5±0.23^{d}$
brown	$2.78±0.1^{a}$	$2.89±0.08^{a}$	$2.71±0.19^{a}$	$2.88±0.08^{a}$	$2.71±0.11^{a}$	$2.64±0.13^{a}$
red	$1.61±0.16^{b}$	$1.56±0.17^{c}$	$1.64±0.23^{c}$	$1.5±0.18^{c}$	$2.07±0.15^{c}$	$2.14±0.21^{bc}$

Different letters within the same column indicate significant difference ($P < 0.05$). SRQ: self-report questionnaire; ET: eye tracking; BMI: body mass index.

Table S7. The shape preferences scores of SRQ or ET measurements of different BMI groups in milk samples.

Stimulus	SRQ-UW	SRQ-NW	SRQ-OW	ET-UW	ET-NW	ET-OW
circle	$2.61±0.14^{a}$	$2.89±0.08^{a}$	$2.79±0.15^{a}$	$2.53±0.16^{a}$	$2.71±0.15^{a}$	$2.83±0.11^{a}$
hexagon	$2.27±0.15^{b}$	$1.94±0.17^{b}$	$1.93±0.16^{b}$	$2.4±0.21^{ab}$	$1.94±0.17^{b}$	$2.17±0.11^{bc}$
pentagon	$1.71±0.16^{c}$	$1.41±0.14^{c}$	$1.5±0.14^{c}$	$2.01±0.19^{bc}$	$1.74±0.19^{c}$	$1.67±0.19^{d}$
square	$1.83±0.19^{c}$	$2.06±0.19^{b}$	$1.71±0.16^{bc}$	$2.13±0.22^{b}$	$2.06±0.21^{b}$	$2.33±0.19^{b}$
triangle	$1.33±0.14^{d}$	$1.56±0.18^{cd}$	$1.29±0.16^{d}$	$1.33±0.16^{d}$	$1.47±0.19^{d}$	$2.17±0.27^{bc}$

Different letters within the same column indicate significant difference ($P < 0.05$). SRQ: self-report questionnaire; ET: eye tracking; BMI: body mass index.

Table S8. The shape preferences scores of SRQ or ET measurements of different BMI groups in coffee samples.

Stimulus	SRQ-UW	SRQ-NW	SRQ-OW	ET-UW	ET-NW	ET-OW
circle	$2.28±0.18^{a}$	$2.5±0.17^{a}$	$2.64±0.17^{a}$	$2.44±0.18^{a}$	$2.63±0.15^{a}$	$2.73±0.13^{a}$
hexagon	$2.17±0.19^{ab}$	$2.17±0.2^{b}$	$2.21±0.21^{b}$	$2.38±0.2^{a}$	$2.31±0.19^{b}$	$2.35±0.15^{b}$
pentagon	$2.06±0.15^{ab}$	$1.88±0.17^{c}$	$1.93±0.2^{bc}$	$1.94±0.16^{b}$	$2.15±0.21^{b}$	$1.91±0.2^{c}$
square	$1.72±0.19^{c}$	$1.99±0.2^{bc}$	$1.86±0.18^{c}$	$1.63±0.17^{c}$	$2.21±0.19^{b}$	$1.91±0.27^{c}$
triangle	$1.56±0.17^{cd}$	$1.67±0.2^{cd}$	$1.36±0.13^{d}$	$1.75±0.21^{bc}$	$1.38±0.15^{d}$	$1.82±0.22^{c}$

Different letters within the same column indicate significant difference ($P < 0.05$). SRQ: self-report questionnaire; ET: eye tracking; BMI: body mass index.
